# PruDensNet: a parameter efficient depthwise separable CNN for MRI-based brain tumor classification

**DOI:** 10.3389/fmed.2026.1743015

**Published:** 2026-05-20

**Authors:** Mithila Arman, Ahnaf Samin, A. K. M. Muzahidul Islam, Md Maruf Rusafi Arnob, Md Jahirul Islam, Ishtiak Al Mamoon

**Affiliations:** 1 Department of Computer Science and Engineering, BRAC University, Dhaka, Bangladesh; 2 Department of Computer Science and Engineering, Ahsanullah University of Science and Technology, Dhaka, Bangladesh; 3 Department of Computer Science and Engineering, United International University, Dhaka, Bangladesh; 4 Department of Computer and Systems Sciences, Stockholm University, Stockholm, Sweden; 5 Department of Computer Science and Engineering, International University of Business Agriculture and Technology, Dhaka, Bangladesh

**Keywords:** attention, augmentation, brain tumor classification, depthwise-separable CNN, MRI, parameter efficiency, reproducibility, SDGs

## Abstract

This study introduces PruDensNet, a parameter-efficient, depthwise-separable convolutional network for magnetic resonance imaging (MRI)-based brain-tumor classification, motivated by compute and latency-sensitive deployments where efficiency can improve throughput and cost. The architecture integrates lightweight channel and spatial attention with Gaussian Error Linear Unit (GELU) activations and a compact global average pooling (GAP)–Dense head, totaling approximately 1.46 M parameters. Architectural effects are isolated from capacity by equalizing parameter budgets across baselines through a no-operation padding mechanism. A reproducible curation pipeline standardizes labels, removes near-duplicates, and applies a stratified split. Training follows a curriculum-regularized recipe combining MixUp, CutMix, random erasing, CLAHE, and label smoothing, with AdamW optimization, warmup-cosine decay, gradient clipping, mixed precision, and test-time augmentation. On a four-class Brain Tumor MRI benchmark (glioma, meningioma, pituitary, no tumor), PruDensNet attains test accuracy 96.05% and validation accuracy 97.27% with competitive per-class metrics, outperforming matched-capacity Convolutional Neural Network (CNN) and Transformer baselines. The results indicate a favorable accuracy-footprint trade-off that can support cost- and latency-sensitive clinical workflows, subject to external validation and hardware-specific benchmarking.

## 1 Introduction

Magnetic resonance imaging (MRI) is the primary modality for non-invasive brain tumor assessment, yet automated analysis remains challenged by heterogeneous protocols, motion, and other artifacts, and substantial inter-tumor variability. Recent challenge iterations (BraTS 2024) have broadened tumor types and refined evaluation regions to better reflect clinical practice (1), raising expectations for robustness and generalizability (2). At the same time, multi-class MRI tumor classification has advanced through self-supervision, hybrid models, and ensembles, though tensions persist among accuracy (3), computational cost, and reproducibility.

Concurrently, efficient-vision research has moved beyond classical MobileNet-style operators toward architectures that co-design blocks (4), attention, and search objectives for edge devices, how depthwise-separable paths and lightweight attention can deliver strong accuracy-latency trade-offs on heterogeneous hardware (5). These trends motivate parameter-efficient backbones for (6) medical imaging that preserve useful inductive biases while remaining deployable on cost-latency-sensitive clinical deployments.

In neuro-oncologic MRI (7), two patterns are evident: hybrid CNN-Transformer and ensemble pipelines often reach high accuracy but with complexity and latency that hinder clinical integration (8), while carefully designed lightweight CNNs remain competitive on common 3/4-class datasets. Attention modules such as SE and CBAM (9), and hardware-friendly long-range interactions enhance discriminative features with minimal overhead (10). This suggests that depthwise-separable stems augmented by lightweight attention can approach the representational power of heavier hybrids while respecting practical memory and latency targets that vary by deployment setting.

Beyond architecture, rigorous methodology is pivotal. Data leakage (slice-level splits, duplicates, patient identity confounds) (11) can inflate metrics and harm reproducibility, underscoring the need for standardized labels, aggressive removal of duplication (12), and subject-wise or file-level disjoint splits. Training recipes that pair MixUp, CutMix with label smoothing and cosine decay (13), plus simple test-time augmentation, improve robustness without increasing inference cost (14). Against this backdrop, this study targets MRI-based brain tumor classification under explicit parameter and memory budgets (15), instantiating a depthwise-separable CNN with lightweight channel-and-spatial attention PruDensNet and evaluating it under a capacity-controlled, leakage-aware protocol.

Target deployment setting: This study does not assume all hospitals are memory-restricted. Instead, this article focuses on scenarios where efficiency directly matters, such as Central Processing Unit (CPU)-only radiology workstations, shared on-prem servers that run multiple services, and low-resource edge installations. In these settings, smaller models can reduce inference time, memory footprint, and operational cost, while still delivering clinically useful accuracy.

The main contribution of this study:

- PruDensNet: a parameter-efficient attention-augmented CNN. This study introduces a lightweight backbone that couples depthwise-separable convolutions with channel (SE) and spatial (CBAM-style) attention, GELU activations, and a compact GAP?Dense head. The design targets an explicit budget of ≈1.46M trainables (~5.6 MB), enabling accurate MRI tumor classification in cost-latency-sensitive deployment contexts.
- Capacity-controlled evaluation via Parameter Target Padding. To ensure fair comparisons, this study matches the total trainable parameters across models by adding a no-op “ParamPad” vector that preserves the forward function while equalizing capacity; this isolates architectural effects from model size.
- Dataset-agnostic, leakage-resistant pipeline. This study contributes an automated data curation stack that, (i) discovers canonical class roots and normalizes label synonyms (ii) removes per-class near-duplicates using an 8 × 8 average-hash (aHash) digested with SHA-1 to curb technical duplicates (iii) performs a stratified file-level 90/10 split with fixed seeds to reduce correlated leakage.
- Curriculum-regularization schedule. This study stages training difficulty with an annealed regimen that ramps MixUp, CutMix, random erasing, CLAHE, and label smoothing mid-course before tapering to zero, improving calibration and generalization without destabilizing early optimization. This study also aligns MixUp and CutMix across paired mini-batches for deterministic, mixed-precision-friendly training.
- Robust and efficient optimization recipe. The system integrates class-imbalance reweighting, AdamW with warmup-cosine decay, global-norm gradient clipping, early stopping, checkpointing, and mixed-precision numerics (float16 compute with float32 logits), plus 5-view test-time augmentation to reduce prediction variance.
- Strong accuracy. On the 4-class Brain Tumor MRI task, PruDensNet attains validation accuracy 97.27% and test accuracy 96.05% with competitive per-class precision, recall, F1, outperforming representative CNN, Transformer, and hybrid baselines under a matched ≈1.4M-parameter constraint.
- Reproducibility by design. This study fixes seeds across the stack, constrains single-process loading to reduce non-determinism, and reports metrics at the best validation checkpoint to ensure repeatability.

## 2 Background study

### 2.1 Convolutional foundations and mobile scaling

Convolutional backbones model images with local, weight-shared filters. A *k* × *k* convolution from *x* to *y* is


$$\begin{array}{l} y_{i,j,c_{o}}=\underset{u,v,c_{i}}{\sum }W_{u,v,c_{i},c_{o}}x_{i+u,j+v,c_{i}} \end{array}$$ (1)

Residual learning stabilizes deep optimization by learning F(x)+x, enabling very deep CNNs, later retuned as ResNet-RS (50) to strong modern baselines via improved scaling and regularization in Hu et al. (16) work.

For on-device efficiency, depthwise separable convolutions factorize the cost from $O\left(k^{2}C_{i}C_{o}HW\right)$ to $O\left(k^{2}C_{i}HW+C_{i}C_{o}HW\right)$. MobileNetV1 (17) adopted depthwise and pointwise operators, MobileNetV2 (18) added inverted residuals with linear bottlenecks expand → depthwise → project preserving information through narrow skips:


$$\begin{array}{l} \text{MBConv}\left(x\right)=x+P_{1\times 1}\left({\text{DW}}_{k\times k}\left(P_{1\times 1}\left(x\right)\right)\right) \end{array}$$ (2)

These ideas remain the template for later mobile hybrids.

### 2.2 Modern ConvNets

ConvNeXt V2 (Tiny) (19) couples a fully-convolutional masked autoencoder (FCMAE) with Global Response Normalization (GRN) (5) to enhance inter-channel competition:


$$\begin{array}{l} \hat{X}=\frac{X}{\epsilon +||X{||}_{2}} \end{array}$$ (3)

$$\begin{array}{l} Y=X⊙\left(\gamma \hat{X}\right)+\beta \end{array}$$ (4)

This co-design lifts pure ConvNets across classification, detection, and segmentation.

InceptionNeXt (Tiny) (20) factorizes large depthwise kernels via multi-branch depthwise paths (square, horiz, vert, identity) fused by 1 × 1 conv, yielding higher throughput at similar accuracy to ConvNeXt. HorNet (Tiny) (21) realizes high-order spatial interactions using recursive gated convolutions *g*^*n*^Conv, capturing long-range effects in a conv framework. NFNet (F0) (22) eliminates BatchNorm via normalizer-free residual blocks with Scaled Weight Standardization and Adaptive Gradient Clipping stabilize training and improve accuracy trade-offs. ResNet-RS (50) (16) shows that strong data-centric training and principled scaling (depth, width, resolution) match newer architectures while improving TPU and GPU efficiency, making it a competitive baseline.

### 2.3 Mobile efficient CNNs

MobileNetV4 (T) (4) introduces the Universal Inverted Bottleneck (UIB), a unified block spanning inverted bottlenecks, ConvNeXt-style MLPs, FFNs, and an Extra-DW variant plus Mobile MQA attention tailored for mobile accelerators, together they deliver Pareto-optimal GPU trade-offs. EfficientNetV2 (B0) (23) combines training-aware NAS, Fused-MBConv, and progressive image-size and regularization schedules for much faster training at similar accuracy. RegNetY (8GF small configs) (24) comes from a low-dimensional design space where stage widths follow an approximately quantized linear rule, yielding simple, fast families competitive across flop regimes.

MobileOne (S0) (25) targets sub-millisecond iPhone latency by training multi-branch blocks that are structurally re-parameterized into single 3 × 3 convolutions at inference, RepVGG-style, reducing memory movement and runtime. GhostNetV2 (26) augments cheap “ghost” features with Decoupled Fully-Connected (DFC) attention, a hardware-friendly long-range mechanism built from 1D FCs along rows and columns, improving accuracy at mobile cost.

### 2.4 Re-parameterized CNNs

RepVGG (A0) (27) trains with parallel identity/1 × 1/3 × 3 branches and fuses them at inference, producing a plain VGG-like stack. Equivalent kernel and bias after fusion:


$$\begin{array}{l} K_{eq}=K_{3\times 3}+pad\left(K_{1\times 1}\right)+K_{id}, \end{array}$$ (5)

$$\begin{array}{l} b_{eq}=b_{3\times 3}+b_{1\times 1}+b_{id}. \end{array}$$ (6)

This retains accuracy with high throughput on commodity hardware.

### 2.5 Vision transformers

Vision Transformer (ViT) (Small) (28) treats images as patch tokens with self-attention:


$$\begin{array}{l} \text{Attn}\left(X\right)=\text{softmax}\left(\frac{QK^{⊤}}{\sqrt{d}}\right)V, \end{array}$$ (7)

achieving strong results when pre-trained at scale.

DeiT III (Small) (29) shows that a simplified, purely supervised recipe (no architectural changes) closes much of the gap to self-supervision and large-data pre-training for ViTs. XCiT (N12) (30) replaces token-wise attention with cross-covariance attention across channels:


$$\begin{array}{l} A=\text{softmax}\left(\frac{Q^{⊤}K}{\sqrt{C}}\right),\;Y=VA^{⊤}, \end{array}$$ (8)

yielding linear complexity in tokens and better scalability to high resolutions.

### 2.6 Hierarchical, windowed, and hybrid transformers

Swin Transformer V2 (Tiny) (31) restricts attention to shifted windows, giving per-block complexity $O\left(HWM^{2}\right)$ for window size *M*, and introduces stable scaling (cosine logit scaling) to train up to multi-billion parameters and megapixel inputs. MaxViT (Tiny) (32) stacks local *block* and global *grid* attentions (“multi-axis”) so the backbone sees global context even in early high-resolution stages near-linear scaling with strong accuracy. CoAtNet0 (33) vertically stacks MBConv (inductive bias and efficiency) with self-attention (global modeling) via relative attention, yielding robust generalization from small to massive data. PVTv2 (B1) (34) builds a pyramid ViT with linear-complexity attention via spatially reduced *K*, *V*, overlapping patch embeddings, and conv FFNs practical for dense prediction.

NextViT (S) (35) mixes Next Convolution Blocks (local) and Next Transformer Blocks (global) for deployment-friendly accuracy, EdgeNeXt (XS) (36) uses split depthwise transpose attention (STDA) to expand receptive fields efficiently for edge devices. TinyViT (37) pretrains compact ViTs via fast distillation from large teachers with a standard distillation loss,


$$\begin{array}{l} L=\left(1-\alpha \right)\text{CE}\left(p_{s},y\right)+\alpha \tau^{2}\text{KL}\left(\frac{p_{t}}{\tau }\|\frac{p_{s}}{\tau }\right), \end{array}$$ (9)

achieving high ImageNet accuracy at a tiny scale.

### 2.7 Mobile efficient hybrids

LeViT (128S) (37) is a ViT “in ConvNet's clothing,” combining hierarchical strides and attention with positional bias. EfficientFormerV2 (S0) (38) jointly searches for parameter trade-offs to reach MobileNet-level speed with transformer token mixing. MobileViT v2 (XS) (39) replaces MHA with separable self-attention that reduces complexity to linear in token count (element-wise ops), markedly improving mobile latency.

### 2.8 MetaFormer MLP-like token mixers

PoolFormer (MetaFormer) (S) (40) argues the *scaffold* (Norm → Token-Mixer → MLP) is the main driver; even a pooling token-mixer works well:


$$\begin{array}{l} y=x+MLP(LN\left(P\left(x\right)-x\right)). \end{array}$$ (10)

This surprisingly strong baseline motivates MetaFormer as a general template.

MLP-Mixer (S) (41) alternates token-mixing and channel-mixing MLPs on patches *X* ∈ ℝ^*N* × *C*^:


$$U=X+\phi (X)W_{t2},\;\phi (X)=LN(X)W_{t1},$$ (11)

$$Y=U+\psi (U)W_{c2},\;\psi (U)=LN(U)W_{c1}.$$ (12)

It shows neither convs nor attention is strictly necessary.

ConvMixer (768) (42) borrows ViT's “patch then factorized mixing” recipe but uses only convs: repeated DWConv (spatial) with residual plus PWConv (channel) mixing after GELU. Despite simplicity, it competes strongly at similar parameter counts.

### 2.9 Attention-like mechanisms inside CNNs

VAN (B0) (43) implements Large-Kernel Attention (LKA), a stack of depthwise and dilated depthwise convs plus 1 × 1 to approximate long-range, content-adaptive interactions with linear-time behavior in CNNs.

FocalNet (Tiny) (44) replaces self-attention with focal modulation, multi-scale depthwise context aggregation, and gated aggregation, producing a modulator *m* that updates features element-wise *y* = *m*⊙*x*. Both deliver transformer-like context without quadratic attention cost.

## 3 Material and methods

In this work, a multi-class medical image classification where the goal is to map an input image *x* ∈ ℝ^*H* × *W* × 3^ to a probability vector over *K* diagnostic categories. Formally, a multi-class medical image dataset $D={\left\{\left(x_{i},y_{i}\right)\right\}}_{i=1}^{N}$ with one-hot labels $y_{i}\in {\left\{0,1\right\}}^{K}$, this study learns parameters θ of a function $f_{\theta }:{\mathbb{R}}^{H\times W\times 3}\to \Delta^{K-1}$ to minimize a class-reweighted, label-smoothed cross-entropy. The custom CNN model architecture is designed to be dataset-agnostic. This ensures the model and training hyperparameters can be applied across brain MRI datasets without manual reconfiguration.

### 3.1 Dataset description

**Figure 1:**
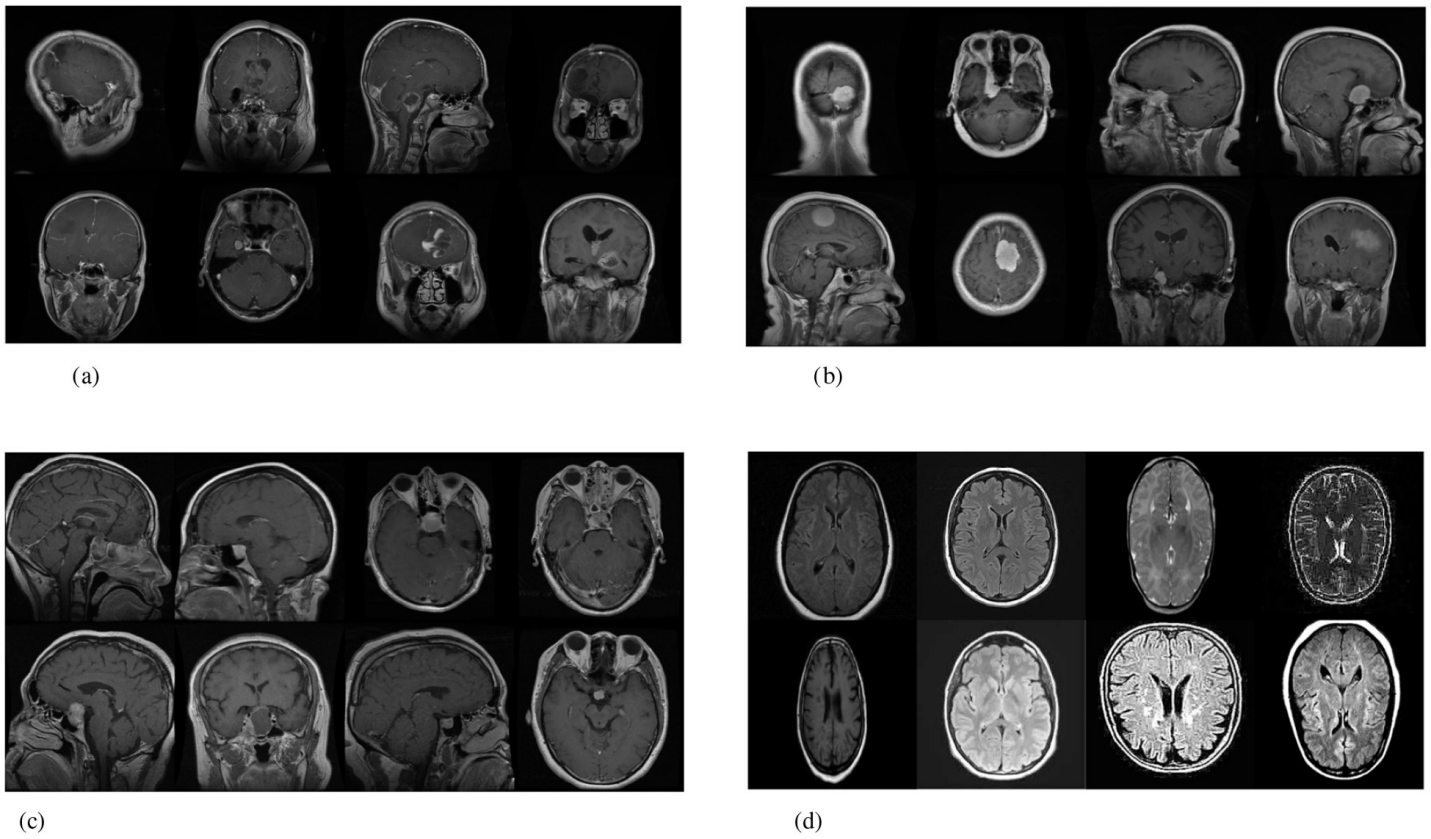
These MRI images illustrate four distinct classes of brain conditions: glioma, meningioma, no tumor, and pituitary tumor. Each set of images presents various views to demonstrate the tumor's characteristics, location, and size within the brain. **(a)** MRI scans of a glioma tumor, showing views. **(b)** MRI images of a meningioma tumor in various planes. **(c)** MRI images showing a pituitary tumor, captured in multiple planes. **(d)** MRI scan showing a healthy brain with no visible tumors.

The Brain Tumor MRI dataset in Figure 1 supports automated image-level brain tumor classification from MRI scans. In this work, we addressed four-class classification (glioma, meningioma, pituitary, and no tumor) and report performance for this image-level task. The dataset does not include longitudinal timelines, tumor staging, or clinical time-to-diagnosis labels. Therefore, our use of detection refers to identifying tumor class from the provided MRI images rather than clinical early-stage detection.

### 3.2 Data discovery, canonicalization, and near-duplicate removal

#### 3.2.1 Automatic source discovery and class normalization

**Figure 2:**
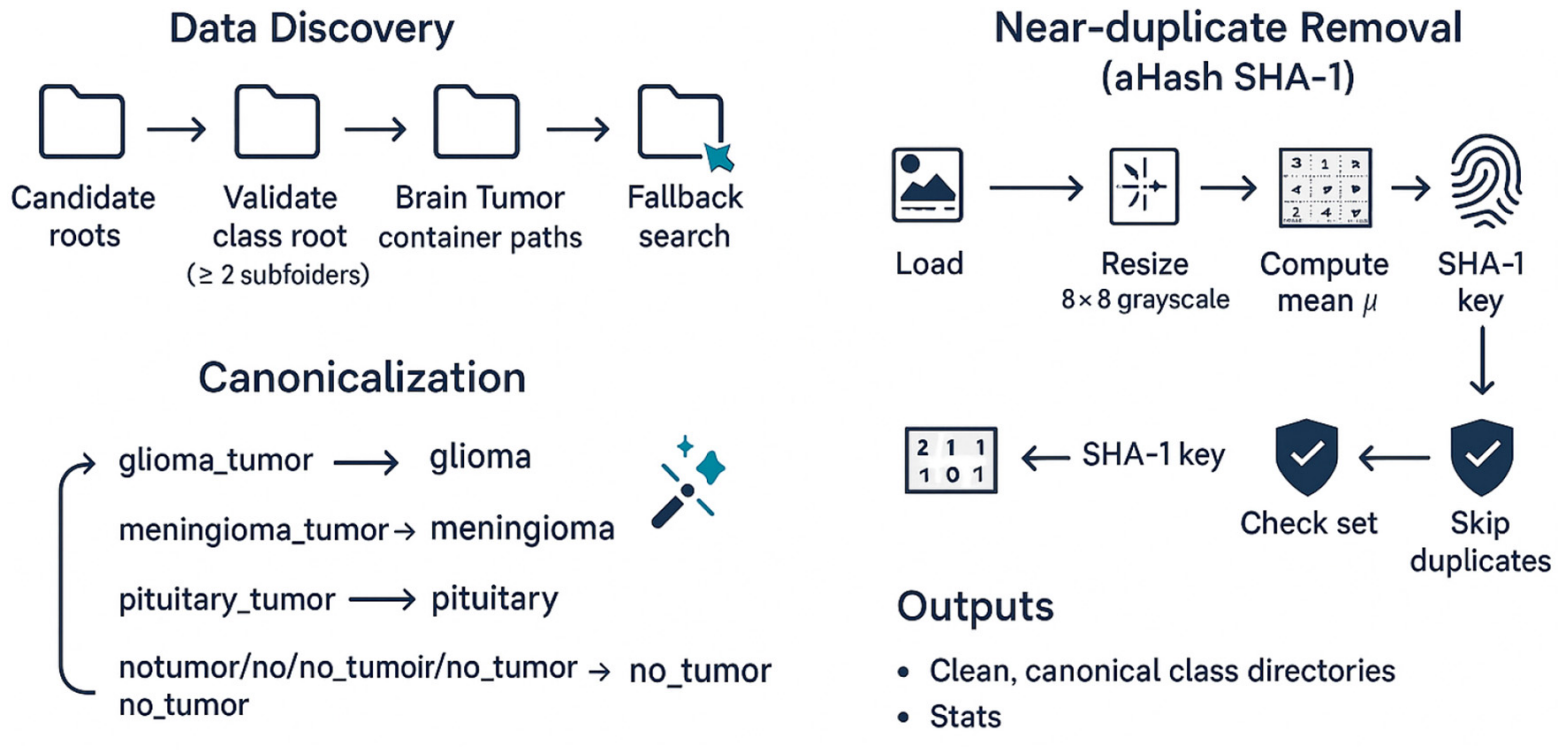
Diagram of data discovery, canonicalization, and near-duplicate removal.

Public medical datasets often differ in the placement of containers (Training/, Testing/) and in the spelling of class names (no_tumour vs. no_tumor). To address this, the code traverses a prioritized list of candidate roots under the data mount and accepts a path *p* as a valid class root if it has ≥2 subdirectories—an assumption that holds for standard class-wise folder layouts. If a brain-tumor dataset is detected, Figure 2, the pipeline further explores known containers and normalizes synonymous class names to a canonical set (glioma_tumor→glioma, notumor→no_tumor). This canonicalization yields consistent label identities across different sources and prevents artificial label fragmentation that would otherwise weaken supervision and confound evaluation. When the dataset provides a predefined Testing/ container, this study treats it as a held-out test set and does not use it for model selection or early stopping.

#### 3.2.2 Per-class near-duplicate removal (average-hash)

Medical image sets frequently contain technical duplicates (identical slices saved with different file names) or near-duplicates (minorly re-compressed copies). This study removes these at the class level using a compact average-hash (aHash) computed from an 8 × 8 grayscale thumbnail. For image *I*, let *G* ∈ ℝ^8 × 8^ be the resized grayscale, $\mu =\frac{1}{64}\underset{u,v}{\sum }G_{uv}$ the mean, and *b*_*uv*_ = 1{*G*_*uv*_>μ}. The 64-bit vector *h* = vec(*b*_*uv*_) is digested with SHA-1 to serve as a key; any subsequent image producing the same key inside a class is ignored. This strict Hamming criterion trades a low false-negative rate for the guarantee that removed items are extremely likely to be redundancies, thereby reducing label leakage across splits and stabilizing accuracy. This yields a flattened class-wise corpus D.

#### 3.2.3 Stratified split

**Figure 3:**
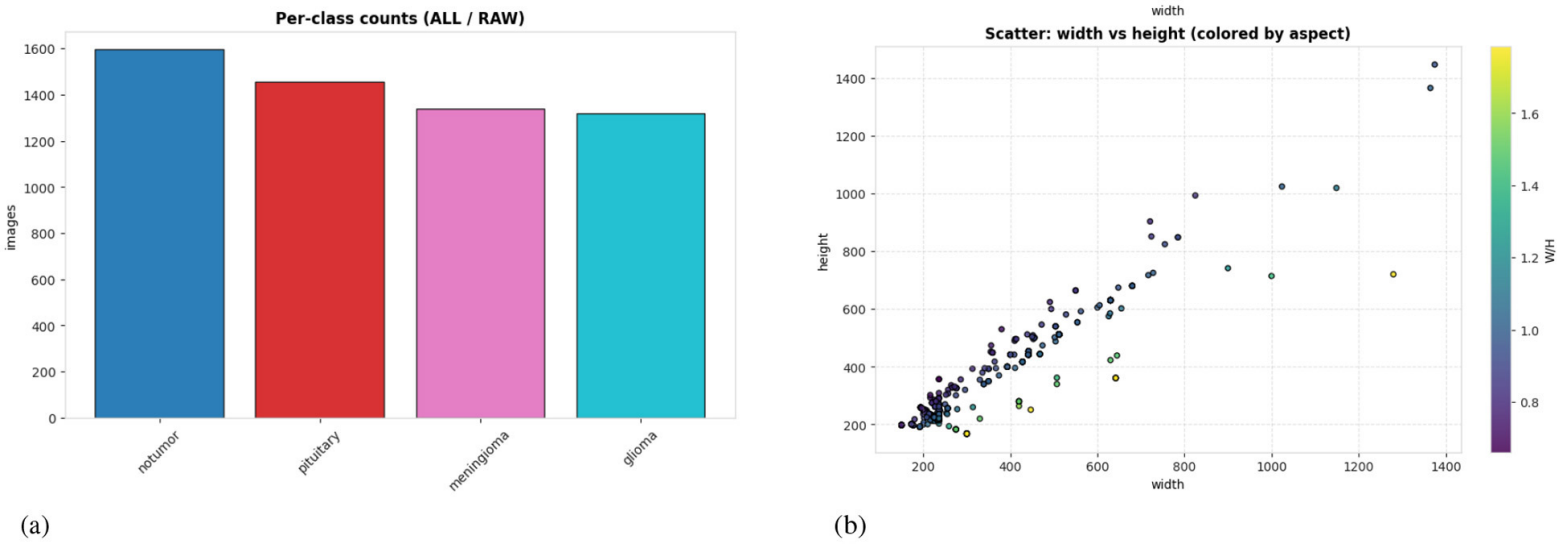
The charts collectively represent key properties of the brain MRI dataset. The bar chart (left) shows the per-class distribution of images across four categories: no tumor, pituitary, meningioma, and glioma, demonstrating a fairly balanced dataset. The scatter plot (right) illustrates the relationship between image width and height, with colors indicating the width-to-height (W/H) aspect ratio, highlighting consistent image proportions with minor variations. **(a)** The bar charts displays the distribution of MRI images across four classes: no tumor, pituitary, meningioma, and glioma. **(b)** The plot illustrates the relationship between image width and height across the dataset.

The dataset provides separate Training/ and Testing/ containers; this study used Testing/ as the held-out test set. Then split Training/ into 90% training and 10% validation using a fixed random seed (42) and stratification by class at the file level. The validation set is used only for early stopping and model selection. All final metrics are reported on the held-out test set. Because the dataset is fairly balanced across classes Figure 3, 10% validation split yields sufficient samples per class for stable early stopping while preserving training data.

Robustness: This benchmark consists of one public dataset, scanner vendor, site, and acquisition protocol metadata not available. Thus, reported performance may not generalize directly across institutions or scanner settings. To quantify the stability of data resampling, this study also presents results across multiple random seeds, summarized in terms of mean1std accuracy and macro-F1 on the held-out test set.

Patient-wise disjointness: The dataset comes in the form of image folders with no patient identifiers. This work pipeline enforces file-level disjointness and will also remove near-duplicates, but cannot fully guarantee that images from the same patient or very correlated acquisitions do not appear in different splits. Hence, this study considers the results of image-level benchmark performance and notes that patient-wise evaluation is necessary for clinical translation.

All images are resized to 256 × 256 pixels and rescaled to the [0, 1] range. This study applies moderate geometric and photometric transformations online using a single-process generator to balance diversity with stability under limited GPU memory. Geometric transforms include rotations within ±10°, width and height shifts up to 8%, shear up to 6°, zoom in [0.8, 1.2], and horizontal flips. Photometric transforms include brightness scaling in [0.92, 1.10]. These operations serve as inductive priors for viewpoint and acquisition variability common to clinical imaging, improving out-of-distribution robustness without drastically altering anatomical content.

CLAHE: When enabled by the annealing schedule, Contrast Limited Adaptive Histogram Equalization is applied to the *L* channel of the CIE-LAB color space, enhancing local contrast while limiting noise amplification:


$$\begin{array}{l} I_{\text{rgb}}\overset{\text{RGB2LAB}}{\to }(L,A,B),\;L^{\prime }=\text{CLAHE}(L),\\ \;I^{\prime }=\text{LAB2RGB}(L^{\prime },A,B). \end{array}$$ (13)

Random erasing: With probability *p*_*e*_, this study masks a randomly sampled rectangle whose area *S*_*e*_~Unif(*s*_min_, *s*_max_)·*HW* and aspect *r*~Unif(*r*_min_, *r*_max_) define


$$\begin{array}{l} H_{e}=\sqrt{S_{e}r} \end{array}$$ (14)

$$\begin{array}{l} \;W_{e}=\sqrt{S_{e}/r} \end{array}$$ (15)

Pixels in the region are replaced by *U*(0, 1) noise, encouraging the network to rely on distributed, pathology-relevant cues rather than spurious local patches.

Standard augmentations are applied:

- Geometric: random rotation θ ∈ [−10°, 10°], width and height shifts up to 0.08 of size, shear up to 6°, horizontal flip.
- Photometric: brightness scaling *b* ∈ [0.92, 1.10].

#### 3.2.4 Curriculum-style augmentation annealing

This study stages the difficulty of training by *annealing* augmentation strengths and label smoothing over epochs *e*:


$$\begin{array}{r} \left(\text{PMixUp},\text{PCutMix},\text{PErase},\text{PCLAE},\epsilon_{\text{LS}}\right)\\ =\{\begin{array}{ll} (0,0,0,0.05), & e<2,\\ (0.15,0.15,0.08,0.06,0.05), & 2\leq e<10,\\ (0.25,0.25,0.12,0.08,0.03), & 10\leq e<36,\\ (0,0,0,0,0), & e\geq 36. \end{array} \end{array}$$ (16)

Early training emphasizes feature acquisition with milder perturbations, mid-training increases regularization (MixUp, CutMix, erasing, CLAHE) to improve calibration and generalization, and late training removes these to consolidate decision boundaries and maximize validation performance.

### 3.3 MixUp and CutMix with aligned batches

**Figure 4:**
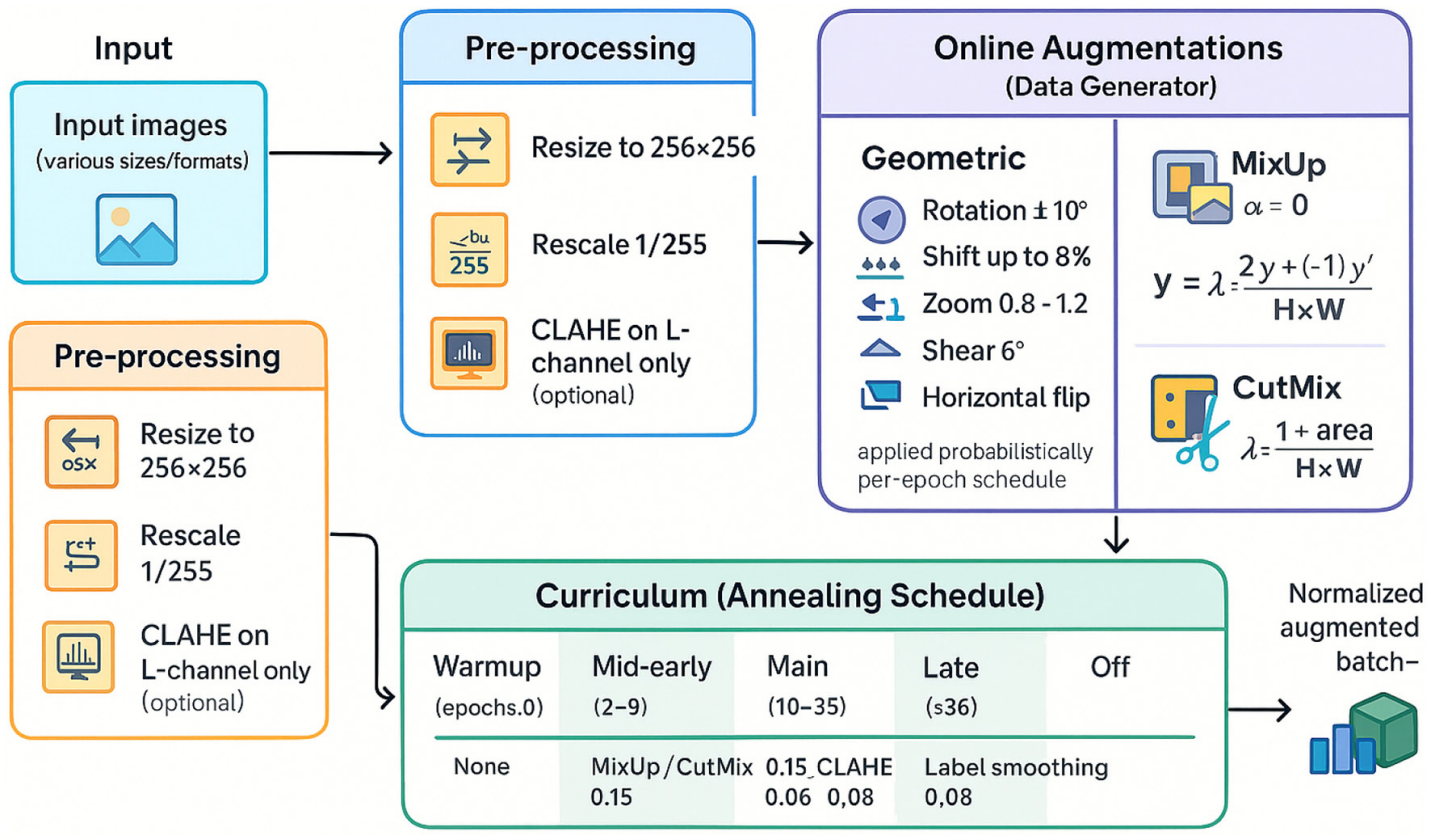
Pre-processing and augmentation with CLAHE, geometric transformations, and curriculum-based augmentation scheduling for enhanced training.

This study implemented a custom sequence that pairs two mini-batches of equal size and applies MixUp or CutMix with probabilities governed in Figure 4. For MixUp, λ~Beta(α, α) with α = 0.3, and each pair (*x, y*), (*x*′, *y*′) yields


$$\begin{array}{l} \tilde{x}=\text{λ}x+\left(1-\text{λ}\right)x^{\prime },\;ỹ=\text{λ}y+\left(1-\text{λ}\right)y^{\prime }. \end{array}$$ (17)

For CutMix, a binary mask *M* ∈ {0, 1}^*H* × *W*^ replaces a random rectangular region of *x* with the corresponding region of *x*′, resulting in a label mixture weighted by the retained area $\text{λ}=\frac{\left|M\right|}{H\times W}$:


$$\begin{array}{l} \tilde{x}=M⊙x+\left(1-M\right)⊙x^{\prime },\;ỹ=\text{λ}y+\left(1-\text{λ}\right)y^{\prime }. \end{array}$$ (18)

Index-aligned pairing avoids re-shuffling overhead and guarantees consistent batch sizes, which is beneficial for mixed-precision stability and deterministic behavior. Two mini-batches ${\left\{\left(x_{i},y_{i}\right)\right\}}_{i=1}^{B}$ and ${\left\{\left(x_{i}^{\prime },y_{i}^{\prime }\right)\right\}}_{i=1}^{B}$ (aligned by index and size), this study samples MixUp and CutMix. The mask area and aspect are restricted to moderate ranges to ensure stable training.

### 3.4 Proposed model architecture

#### 3.4.1 Overview

**Figure 5:**
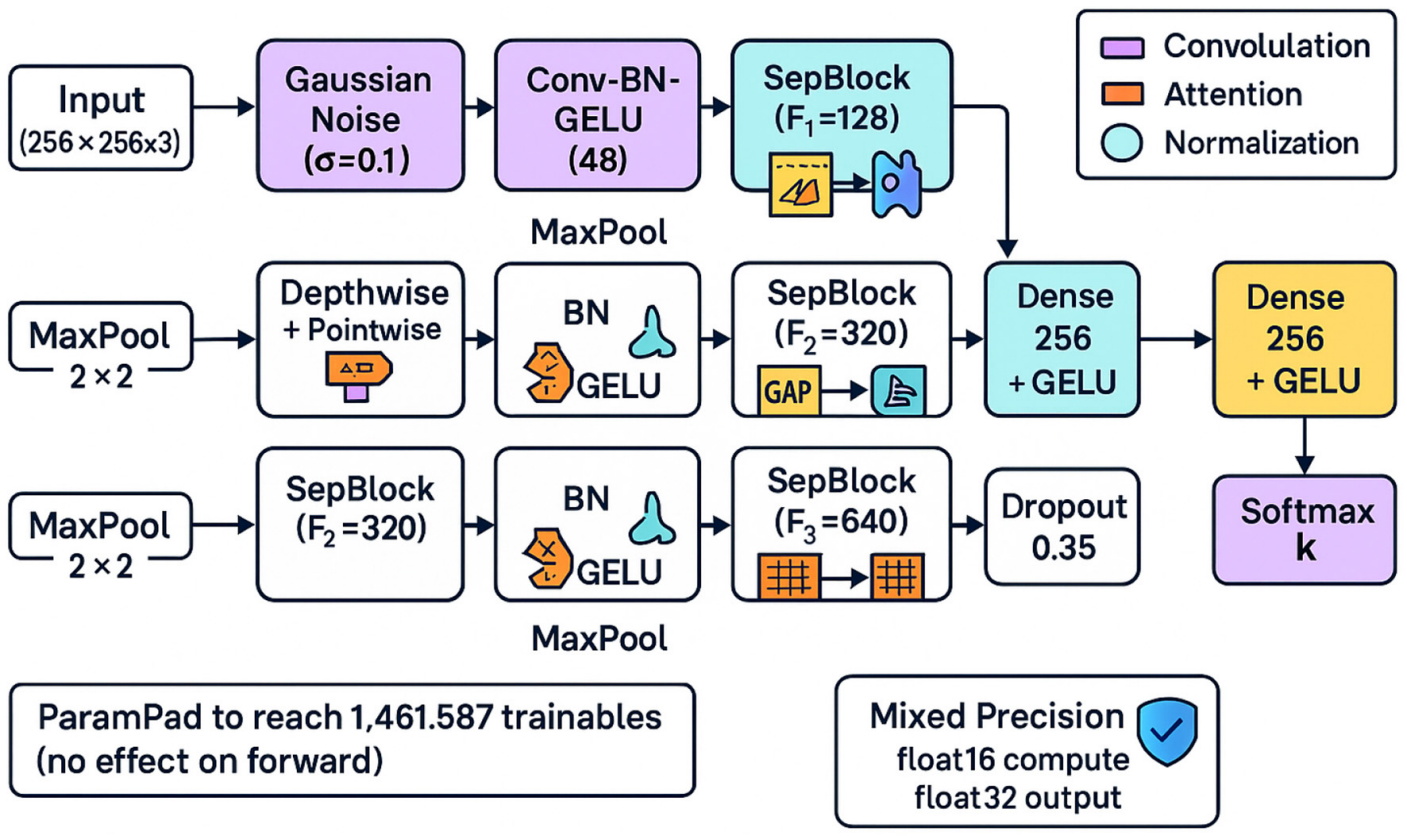
Our proposed model architecture with SepBlocks and mixed precision.

The model is a lightweight attention-augmented CNN optimized for stability and efficiency. In Figure 5 It begins with Gaussian noise injection and two Conv-BN-GELU layers, followed by three depthwise-separable blocks at increasing channel widths *F*_1_ = 128, *F*_2_ = 320, *F*_3_ = 640. Each block integrates channel and spatial attention, and blocks are separated by 2 × 2 max pooling. A global average pooling (GAP) layer aggregates spatial information, feeding a 256-unit GELU-activated bottleneck with dropout *p* = 0.35, then a *K*-way softmax.

A lightweight attention-augmented Custom CNN model architecture with depthwise separable convolutions, channel and spatial attention, and global pooling:


$$\begin{array}{c} x\overset{\text{GaussianNoise}}{\to }\overset{\text{ConvBNGELU}\times 2}{\to }\overset{\text{Pool}}{\to }\overset{\text{SepBlock}(F_{1})}{\to }\overset{\text{Pool}}{\to }\overset{\text{SepBlock}(F_{2})}{\to }\\ \overset{\text{Pool}}{\to }\overset{\text{SepBlock}(F_{3})}{\to }\overset{\text{Pool}}{\to }\overset{\text{GAP}}{\to }\\ \overset{\text{Dense}(256)+\text{GELU}+\text{Dropout}}{\to }\overset{\text{Softmax}(K)}{\to } \end{array}$$

#### 3.4.2 Depthwise separable convolution

Separable convolutions reduce parameters and multiply-accumulate operations by decomposing a standard *k* × *k* convolution with *C*→*C*′ channels (cost *k*^2^*CC*′) into a depthwise part (*k*^2^*C*) followed by a pointwise 1 × 1 projection (*CC*′). This improves training speed and memory footprint while preserving representational capacity, particularly when combined with attention mechanisms that selectively emphasize informative channels and locations. For input *X* ∈ ℝ^*H* × *W* × *C*^ and kernel size *k*, parameter count reduces from *k*^2^*CC*′ to *k*^2^*C*+*CC*′.

#### 3.4.3 GELU activation

This study adopts the Gaussian Error Linear Unit for its smooth gating behavior and strong empirical performance in vision models:


$$\begin{array}{l} \text{GELU}\left(z\right)=\frac{1}{2}z\left(1+\tanh\left[\sqrt{\frac{2}{\pi }}\left(z+0.044715z^{3}\right)\right]\right). \end{array}$$ (19)

GELU encourages gradient flow for small negative activations relative to ReLU, which can be advantageous in shallow bottlenecks and under label smoothing.

#### 3.4.4 Channel attention (squeeze-and-excitation)

Given feature map *U* ∈ ℝ^*H* × *W* × *C*^, GAP yields *z* ∈ ℝ^*C*^ with


$$\begin{array}{l} z_{c}=\frac{1}{HW}\underset{i,j}{\sum }U_{ijc}. \end{array}$$ (20)

The excitation pathway performs a bottleneck transformation with reduction ratio *r* = 16:


$$\begin{array}{l} s=\sigma \left(W_{2}\delta \left(W_{1}z\right)\right),\;W_{1}\in {\mathbb{R}}^{r\times C},\;W_{2}\in {\mathbb{R}}^{C\times r}, \end{array}$$ (21)

where δ is ReLU and σ is sigmoid. The re-scaling Û_*ijc*_ = *U*_*ijc*_·*s*_*c*_ emphasizes diagnostically salient channels (tumor textures) while suppressing noise.

#### 3.4.5 Spatial attention (CBAM-style)

Complementing channel attention, spatial attention aggregates across channels via average and max pooling to produce two maps $M_{\text{avg}},M_{\text{max}}\in {\mathbb{R}}^{H\times W}$. Their concatenation is convolved with a 7 × 7 filter and squashed through a sigmoid to form *S*:


$$\begin{array}{l} S=\sigma \left({\text{Conv}}_{7\times 7}\left(\left[M_{\text{avg}};M_{\text{max}}\right]\right)\right), \end{array}$$ (22)

$$\begin{array}{l} {Û}_{ijc}=U_{ijc}\cdot S_{ij}. \end{array}$$ (23)

This mechanism allows the network to focus spatially on relevant loci (lesion regions) regardless of channel identity.

#### 3.4.6 Global pooling head and dropout

GAP converts the final feature tensor to a single vector by averaging over spatial dimensions, which–unlike fully connected spatial layers–reduces parameters and overfitting risk. A dense layer with 256 units and GELU non-linearity captures cross-channel interactions before classification. Dropout with a rate of 0.35 regularizes this compact head, which is particularly effective when coupled with label smoothing and MixUp and CutMix.

#### 3.4.7 Parameter target padding

To enable capacity-controlled comparisons and ablations, the base architecture is sized to be at or below a fixed budget P^*^ = 1, 461, 587 trainables. If the realized count $P_{\text{base}}<P^{*}$, this study adds a ParamPad layer with trainable vector $p\in {\mathbb{R}}^{P^{*}-P_{\text{base}}}$ that is multiplied by zero in the forward pass:


$$\begin{array}{l} y^{\prime }=y+0\cdot \underset{j}{\sum }p_{j}. \end{array}$$ (24)

This keeps gradients and numerics unchanged while matching the exact parameter target, isolating performance differences to architectural design rather than capacity.

#### 3.4.8 Mixed precision numerics

This study trains with TensorFlow's mixed_float16 policy to accelerate computation and reduce memory while keeping the classifier output in float32 to preserve loss stability. Mixed precision is especially beneficial with small batch sizes on commodity GPUs by allowing higher-resolution inputs and larger models without out-of-memory errors.

### 3.5 Optimization and regularization

#### 3.5.1 Class imbalance reweighting

**Figure 6:**
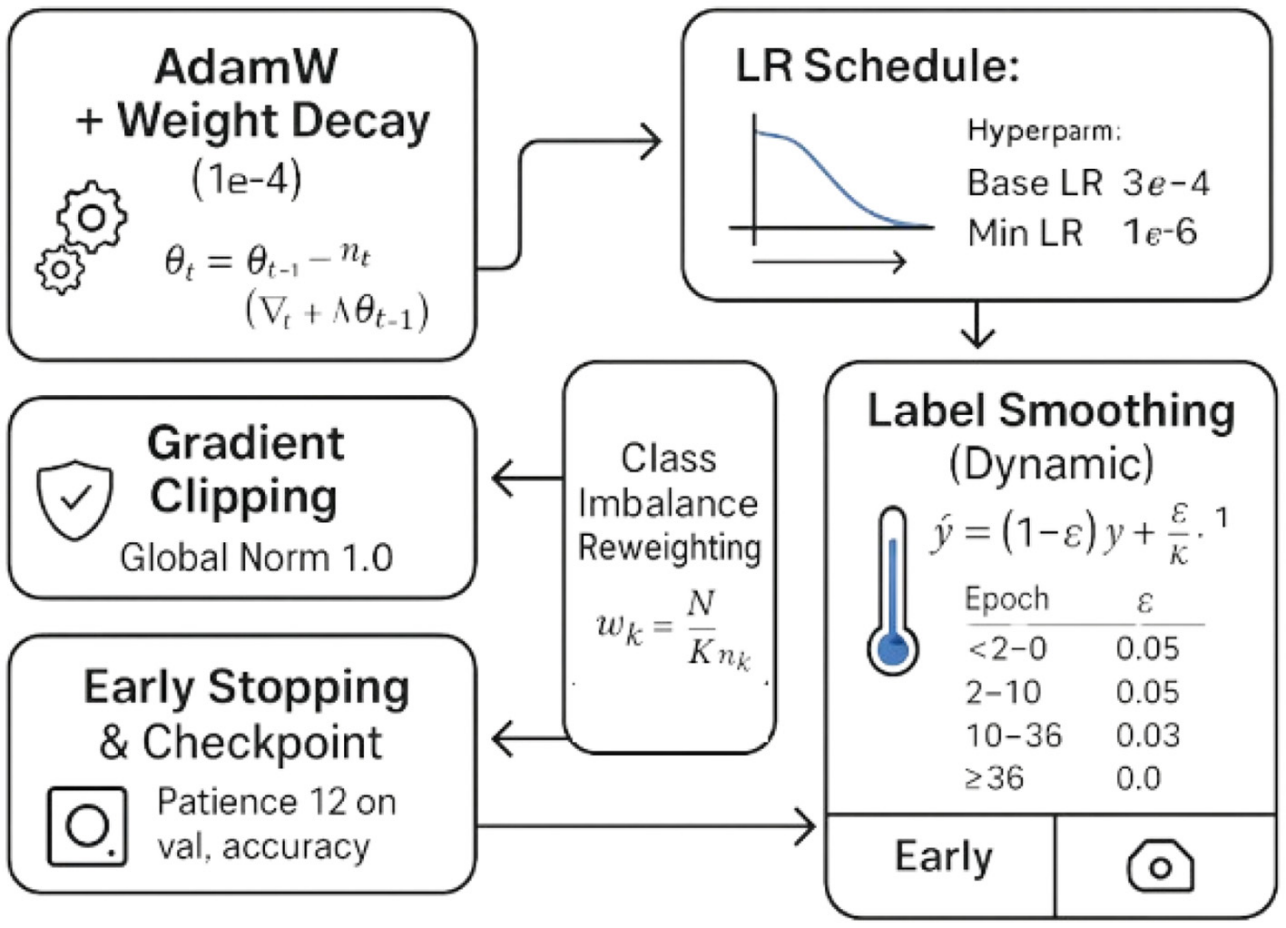
Optimization and Regularization Techniques for Model Training.

Class frequencies *n*_*k*_ can vary substantially across medical categories. This study weighs each class by $w_{k}=\frac{N}{Kn_{k}}$, so the expected contribution per class to the total loss is approximately uniform. This simple inverse-frequency heuristic reduces bias toward common classes and improves minority-class recall without modifying the data distribution itself in Figure 6.

#### 3.5.2 Label smoothing with dynamic schedule

Label noise and strong augmentations can yield overconfident predictions. This study, therefore, replaces hard targets *y* with smoothed targets:


$$\begin{array}{l} ỹ=\left(1-\epsilon \right)y+\frac{\epsilon }{K}1, \end{array}$$ (25)

where ϵ follows the epoch-wise schedule in The training loss remains the categorical cross-entropy $L_{\text{CE}}\left(ỹ,\hat{p}\right)=-\underset{k}{\sum }{ỹ}_{k}\log{\hat{p}}_{k}$, which empirically improves calibration and robustness to MixUp and CutMix label interpolation.

#### 3.5.3 AdamW with warmup-cosine schedule and gradient clipping

This study optimizes with AdamW (decoupled weight decay λ = 10^−4^) and global-norm clipping (||***g***||_2_ ≤ 1) to prevent gradient spikes when augmentations intensify. The learning rate η_*t*_ uses linear warmup over the first *T*_*w*_ steps and cosine decay over *T*_*d*_ steps with floor η_min_:


$$\begin{array}{l} \eta_{t}=\eta_{\text{min}}+\left(\eta_{0}-\eta_{\text{min}}\right)\cdot \min\left(1,\frac{t}{T_{w}}\right)\cdot \left(1+\cos\left(\frac{\pi \left(t-T_{w}\right)}{T_{d}}\right)\right). \end{array}$$ (26)

Warmup mitigates instability in early epochs with mixed precision and strong data augmentation, while the cosine schedule encourages smooth convergence and slight late-stage exploration.

#### 3.5.4 Early stopping and checkpointing

Training proceeds for up to 50 epochs with early stopping on accuracy (patience 12). The best model by accuracy is checkpointed and restored for evaluation. This protocol controls overfitting and ensures reported metrics correspond to the empirically strongest validation point rather than the final epoch.

### 3.6 Inference-time Test-Time Augmentation (TTA)

At evaluation, this study averages predictions over *T* = 5 deterministic transforms: identity, horizontal flip, rotations of ±8°, and a mild gamma adjustment. For input *x*, the final probability is:


$$\begin{array}{l} \tilde{p}=\frac{1}{T}\overset{T}{\underset{t=1}{\sum }}f_{0}\left(\tau_{t}\left(x\right)\right),\;ŷ=\arg\underset{k}{\max}{\tilde{p}}_{k}. \end{array}$$ (27)

This reduces variance associated with borderline cases whose predictions are sensitive to minor orientation or contrast changes, typically improving both accuracy and calibration on the held-out test data.

### 3.7 Evaluation metrics

This study reports overall accuracy $\text{Acc}=\frac{1}{M}\overset{M}{\underset{i=1}{\sum }}1\left\{{ŷ}_{i}=y_{i}\right\}$ on the held-out test split, along with a confusion matrix *C* ∈ ℕ^*K* × *K*^ and per-class precision, recall, and F1:


$$\begin{array}{l} {\text{Prec}}_{k}=\frac{C_{kk}}{\sum_{j}C_{jk}},\;{\text{Rec}}_{k}=\frac{C_{kk}}{\sum_{j}C_{kj}},\;{\text{F1}}_{k}=\frac{2\cdot {\text{Prec}}_{k}\cdot {\text{Rec}}_{k}}{{\text{Prec}}_{k}+{\text{Rec}}_{k}}. \end{array}$$ (28)

Macro and weighted averages summarize class-wise behavior across imbalanced datasets, providing a nuanced view beyond accuracy, especially important when minority classes are clinically significant. The validation split is used only for early stopping and checkpoint selection.

### 3.8 Reproducibility and systems

All experiments were run on an NVIDIA Tesla P100 GPU in a Kaggle-style environment, mixed precision and GPU memory growth were enabled for stability and to avoid large pre-allocations. This study fixes random seeds for Python, NumPy, and TensorFlow, and enables GPU memory growth to avoid large pre-allocations that can trigger OOMs on shared accelerators. Data loading remains single-process to reduce non-determinism associated with multi-worker prefetching in some backends. Mixed precision is enabled globally, while the classification logits/softmax are computed in float32 to stabilize the loss. Together, these practices limit run-to-run variance and make experiments repeatable on Kaggle-style execution environments.

### 3.9 Complexity and capacity control

Let $P\left(\theta \right)=\underset{l}{\sum }\prod_{\text{dshape}\left(W_{l}\right)}d$ denote trainable parameters. This study designs widths so that $P\left(\theta_{\text{base}}\right)\leq P^{*}=1,461,587$. If $P\left(\theta_{\text{base}}\right)<P^{*}$, the ParamPad layer increases the count to exactly P^*^ without changing the input–output mapping:


$$\begin{array}{l} f_{\theta ,\text{padded}}\left(x\right)=f_{\theta }\left(x\right). \end{array}$$ (29)

This ensures comparisons across ablations are attributable to architectural choices rather than capacity differences, a common confound in model development.

Yet equalizing parameters does not imply equalizing compute. Thus, this study also includes computation cost and deployment-relevant performance: (i) MACs FLOPs for a single 256 × 256 input, (ii) peak GPU memory during inference, and (iii) inference latency (batch=1) on the target hardware. This study reports latency as the median over N runs after a warm-up phase, using the same preprocessing and TensorFlow execution settings for all models.

### 3.10 Key hyperparameter implementation

The full system couples a modest-resolution input (256^2^), a batch size of eight suited to GPU memory constraints, and a depthwise-separable backbone augmented with SE and spatial attention. The training recipe intentionally layers complementary regularizers—MixUp, CutMix, random erasing, CLAHE, and label smoothing—under a time-varying schedule that begins conservatively and intensifies mid-course before tapering to zero. Optimization uses AdamW with global-norm clipping and a warmup-cosine learning-rate schedule starting at 3 × 10^−4^ with a floor of 10^−6^. Class imbalance is countered by inverse-frequency weighting so that rare classes meaningfully influence the objective. Early stopping on accuracy governs training duration, and the best checkpoint is evaluated using 5-view TTA to reduce prediction variance. This combination yields a compute-efficient, capacity-controlled, and reproducible pipeline that is robust to dataset idiosyncrasies encountered in real-world medical imaging.

- Input resolution: 256 × 256.
- Batch size: 8. W
- Augmentations: Rotations ±10°, flips, shifts ≤ 0.08, shear 6°, zoom ±20%, brightness [0.92, 1.10], CLAHE/erasing (annealed), MixUp/CutMix (annealed, α = 0.3).
- Attention: SE (*r* = 16), spatial gate (7 × 7).
- Head: GAP → Dense(256, GELU) → Dropout(0.35) → Softmax(*K*).
- Optimizer: AdamW (λ = 10^−4^, global clip-norm = 1.0).
- LR schedule: armup-cosine ($\eta_{0}=3\times 10^{-4}$, $\eta_{\text{min}}=10^{-6}$, warmup = 10% of steps).
- Loss: Cross-entropy with dynamic label smoothing ϵ(*e*).
- Class weighting: $w_{k}=\frac{N}{Kn_{k}}$.
- TTA: 5 augments (mean probability).
- Stopping: Patience 12 on val-accuracy; best checkpoint restored.

## 4 Experimentation and results

### 4.1 Algorithmic framework of proposed model

In Algorithm 1 establishes a leakage-resistant dataset by automatically locating the class root, canonicalizing labels to glioma, meningioma, pituitary, no tumor, filtering per-class near-duplicates via an 878 grayscale average-hash digested with SHA-1, and then performing a fixed-seed stratified train and validation split. In Algorithm 2 trains a parameter-controlled depthwise-separable CNN with SE and spatial attention blocks at progressively wider stages, using inputs, batch size, AdamW with warmup-cosine decay and global-norm clipping, inverse-frequency class weighting, and a curriculum that anneals regularizers MixUp, CutMix, random erasing, CLAHE, and dynamic label smoothing from zero in early epochs to a mid-course peak and back to zero, with early stopping on accuracy and mixed-precision compute. In Algorithm 3 reloads the best checkpoint and performs variance-reduced evaluation via five deterministic test-time transforms, averaging probabilities to yield final predictions before reporting accuracy, confusion matrix, and per-class precision, recall, F1, capacity-matched training, and robust inference in a clinically mindful, reproducible workflow.

Algorithm 1 **Data curation, near-duplicate removal, and stratified split (90/10).**

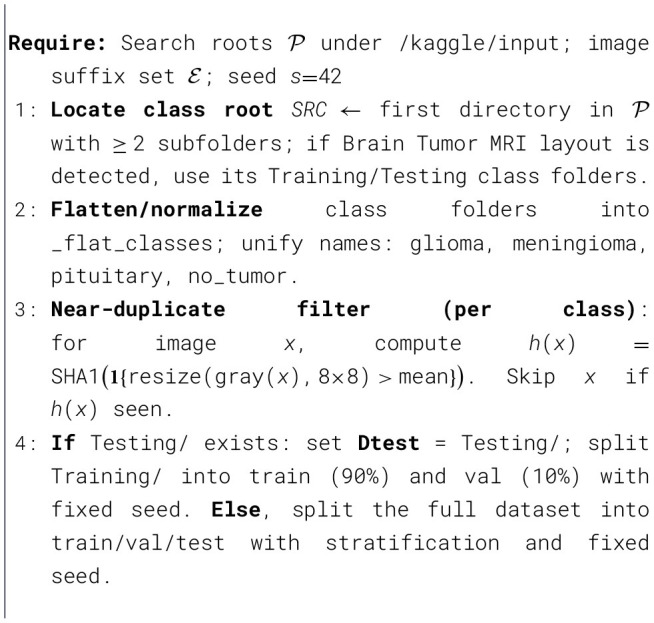

Algorithm 2 **Training with SE+spatial attention SepConv, annealed aug, warmup-cosine.**

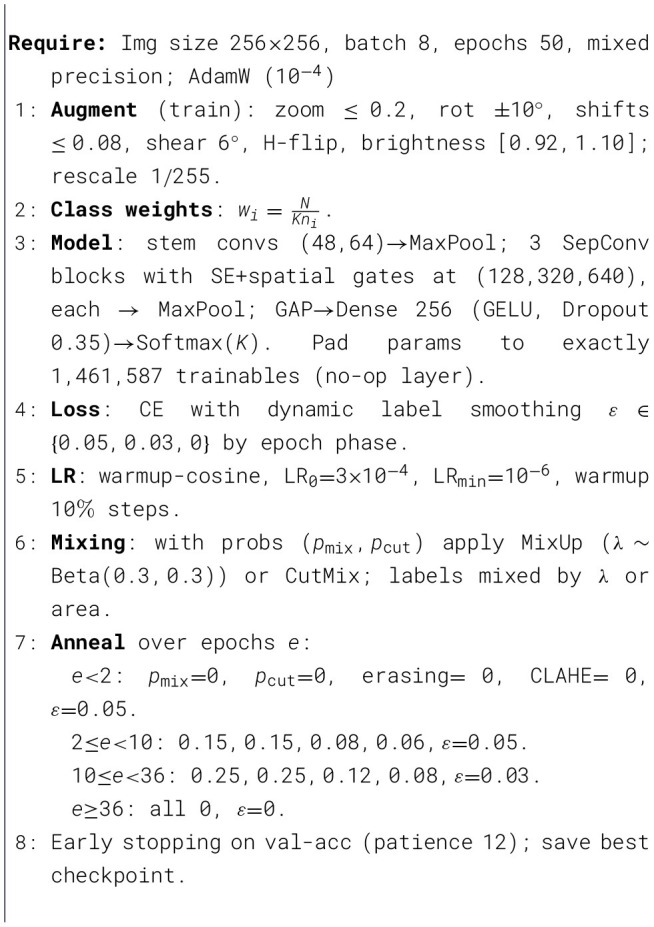

Algorithm 3 **Evaluation with 5 × Test-Time Augmentation (TTA).**

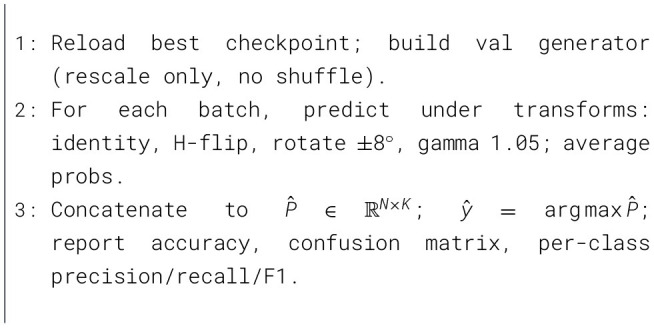

In Table 1, the experimental setup is specified end-to-end: dataset, split policy, fixed seed, input resolution, augmentation suite, architecture family, and optimization and loss choices. The parameter ≈1.4M, ~5.6 MB is fixed to enable capacity-controlled comparisons. Batch size, schedule, and stopping criteria are explicitly recorded for reproducibility. Evaluation metrics include overall accuracy plus per-class precision, recall, F1 with TTA at inference. This table serves as the study's reproducibility contract. Table 2, compares PruDensNet against matched-capacity CNNs, Transformers, hybrids, and MLP-like baselines (all ≈1.4M params). PruDensNet achieves test accuracy 96.05% and Validation accuracy 97.27% with low loss, outperforming popular small backbones under the same parameter budget. Several strong baselines trail slightly despite a similar footprint. Others degrade markedly when downsized, underscoring the value of the proposed design for small models. The table isolates architecture, not size, as the differentiator. EfficientFormerV2-S0 and RegNetY-8GF are competitive, while some light ViTs and metaformers trail under this capacity constraint. The standardized parameter cap ensures fairness by isolating architectural design rather than sheer size. The breadth of baselines spans modern convnets, hybrids, and transformers. The table evidences that the core CNN design can excel at tiny footprints.

**Table 1:** Experimental setup.

Aspect	Configuration
Dataset	Brain Tumor MRI (4 classes: glioma, meningioma, pituitary, no tumor).
Split	Split Predefined Testing/ used as held-out test set; Training/ split stratified 90/10 train/val at file level (fixed seed).
Input size & scaling	Images resized to 256 × 256; values scaled to [0, 1].
Augmentations	Rotations ±10°, shifts ≤ 0.08, shear 6°, zoom [0.8, 1.2], horizontal flip, mild brightness jitter.
Architecture	Lightweight CNN with depthwise separable conv blocks and attention; GAP head with dense classifier.
Parameters	≈1.4M trainable parameters (model size ~5.6 MB).
Loss	Categorical cross-entropy (label smoothing).
Optimizer	AdamW with weight decay; gradient clip-norm = 1.0.
LR schedule	Warmup–cosine; min LR 10^−6^, base LR 3 × 10^−4^.
Batch size	8.
Training duration	Up to 50 epochs with early stopping on accuracy.
Evaluation	Accuracy and per-class precision/recall/F1; confusion matrix.
Inference	Test-time augmentation (flips/rotations); average probabilities.

**Table 2:** Model comparison (PruDensNet (Our model): ≈ 1.4M params, ~ 5.6 MB).

Model	Accuracy	Loss
**PruDensNet (ours)**	**0.9727**	**0.1027**
MobileNetV4 T	0.7235	0.9451
EfficientNetV2 B0	0.9501	0.1409
ConvNeXt V2 Tiny	0.7879	0.5978
MaxViT Tiny	0.9002	0.2399
CoAtNet0	0.9044	0.4446
Swin Transformer V2 Tiny	0.9293	0.4705
DeiT III Small	0.9418	0.3070
Vision Transformer (ViT) Small	0.6486	1.0678
RegNetY 8GF	0.9563	0.4415
LeViT (128S)	0.9501	0.1981
EfficientFormerV2 S0	0.9638	0.1885
MobileViT v2 XS	0.8420	0.2921
TinyViT (21M)	0.9085	0.1548
FocalNet Tiny	0.7817	0.5984
VAN (Visual Attention Network) B0	0.7817	0.5984
PVTv2 B1	0.9252	0.4463
NextViT S	0.9127	0.1777
EdgeNeXt XS	0.8607	0.5961
PoolFormer (MetaFormer) (S)	0.5426	0.9740
XCiT (N12)	0.8565	0.4002
RepVGG (A0)	0.6881	0.6804
NFNet (F0)	0.9522	0.4230
GhostNetV2	0.5925	0.9740
ConvMixer (768)	0.9459	0.1221
MLP-Mixer (S)	0.6112	1.0162
ResNet-RS (50)	0.9501	0.9709
InceptionNeXt (Tiny)	0.9584	0.5084
MobileOne (S0)	0.5509	1.3208
HorNet (Tiny)	0.8462	0.1733

### 4.2 Result analysis and performance

**Figure 7:**
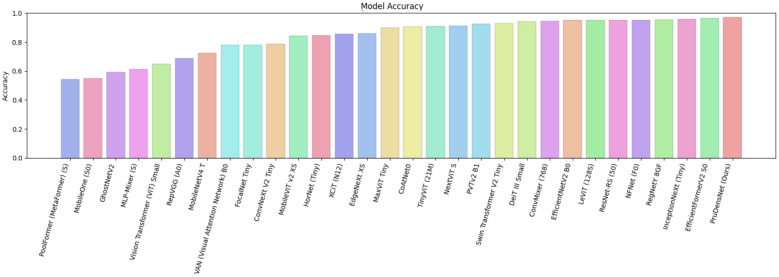
Model accuracy comparison, a bar chart showing the classification accuracy achieved by each evaluated architecture on the same test setting. Models are ordered from lowest to highest accuracy (left to right). PruDensNet (ours) attains the best accuracy among all compared methods.

**Figure 8:**
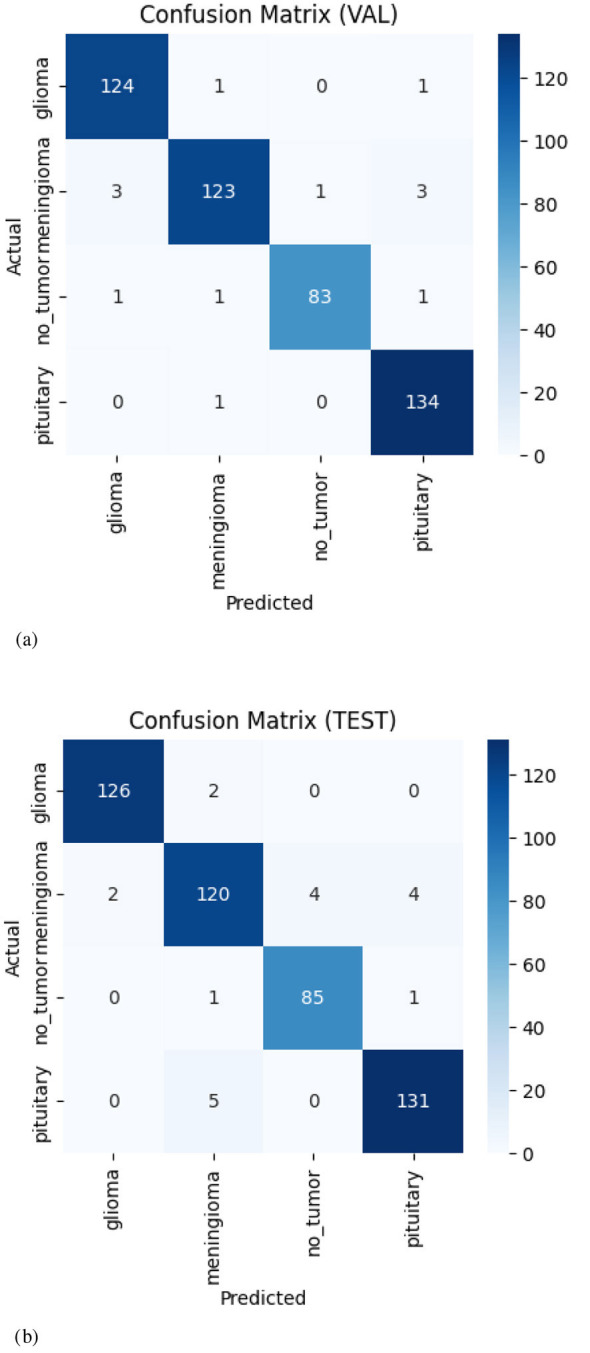
Confusion matrices for the four-class brain tumor classifier (glioma, meningioma, no_tumor, pituitary) on the validation and test splits. Most predictions fall along the diagonal, indicating high correct classification across classes, with only a small number of misclassifications between a few class pairs. **(a)** Confusion matrix on validation set (VAL). Rows shows the actual class and columns shows the predicted class. **(b)** Confusion matrix on test set (TEST). Rows shows the actual class and columns shows the predicted class.

As shown in Figure 7, PruDensNet achieves the highest accuracy among all evaluated architectures. In Figure 8, a confusion matrix summarizes for still works for the four-class brain tumor classifier (glioma, meningioma, no tumor, pituitary) for both the validation and test splits. Most predictions lie above or below the diagonal, which indicates good class-wise accuracy, with very few misclassifications between a few class pairs.

Calibration and reliability analysis: However, Table 3 for safety-critical decision support, accuracy is not sufficient; models must also generate reliable estimates of confidence. This study therefore reports Expected Calibration Error (ECE) in terms of equal-width confidence bins and the Brier score. Reliability diagrams that are used as well give a visual representation of the correlation between estimated confidence and actual accuracy. Since the training framework involved label smoothing and strong regularization, this article expects better calibration than unregularized baselines. Nevertheless, calibration will need to be confirmed empirically on the held-out test set. Table 4 comparison of baselines model size, computational cost, peak GPU memory, and inference latency.

**Table 3:** Calibration performance on validation and test sets (4 classes).

Split	*N*	ECE ↓	Brier Score ↓
Val	477	0.009690	0.058287
Test	481	0.021510	0.070982

**Table 4:** Comparison of six strong baselines in terms of model size, computational cost, peak GPU memory, and inference latency (Tesla P100).

Model	Params (M)	MACs (G)	FLOPs (G)	Peak GPU Mem (MB)	Latency (ms)
PruDensNet (ours)	1.46159	3.68751	7.37503	4690.53	12.031
MobileNetV4 T	3.7740	0.2410	0.4819	177.025	4.6233
EfficientNetV2 B0	7.1397	0.8468	1.6936	193.530	8.9015
ConvNeXt V2 Tiny	28.6355	5.8193	11.6386	292.222	8.3387
Swin Transformer V2 Tiny	28.3472	4.3709	8.7418	522.529	14.8396
RegNetY 8GF	39.1801	10.3987	20.7974	565.389	11.4717
MaxViT Tiny	30.9165	5.3330	10.6660	430.379	25.2587

**Figure 9:**
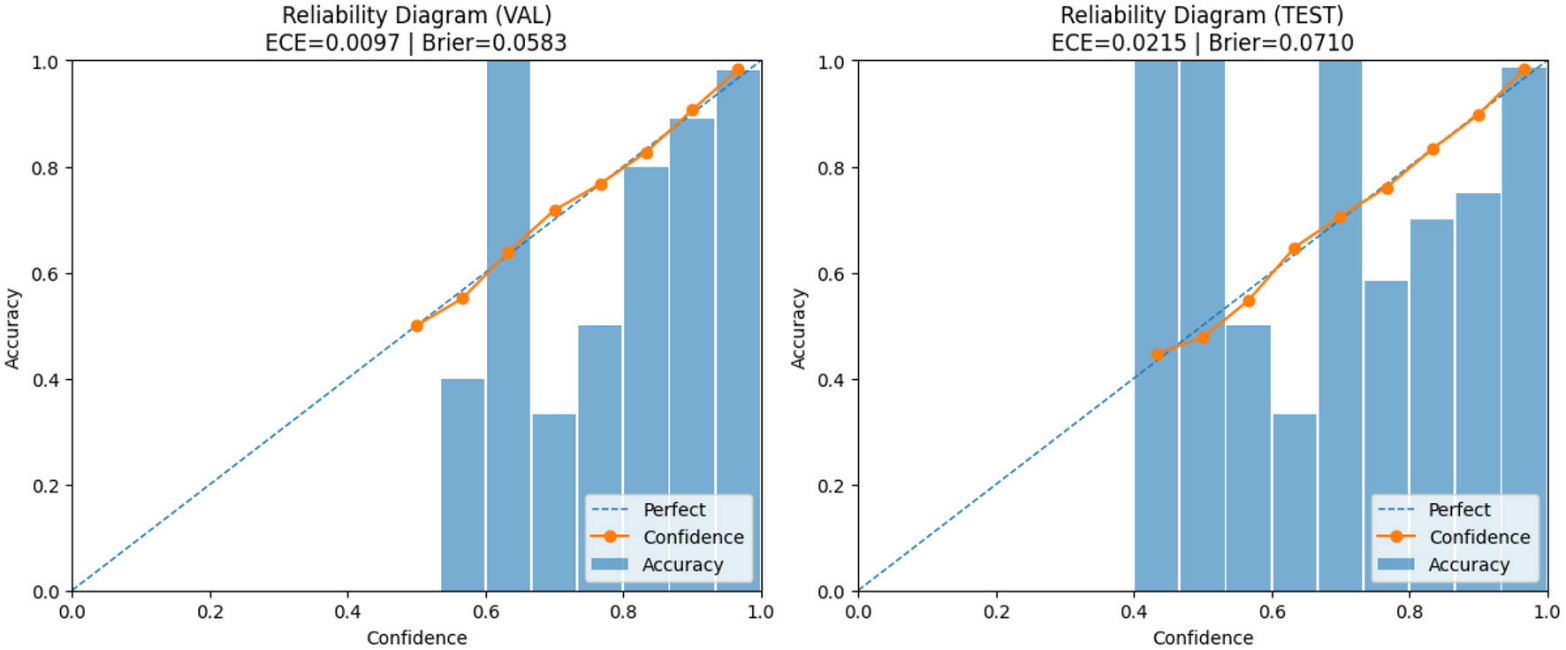
Reliability diagrams on the validation **(Left)** and test **(Right)** sets for a 4-class classifier. The dashed diagonal indicates perfect calibration; orange markers show mean confidence per bin, and blue bars show empirical accuracy.

In Figure 9, compare the reliability diagrams for a 4-class classifier on the validation (left) and test (right) sets. Dashed diagonal represents perfect calibration, orange markers are mean confidence per bin, and blue bars indicate empirical accuracy.

**Figure 10:**
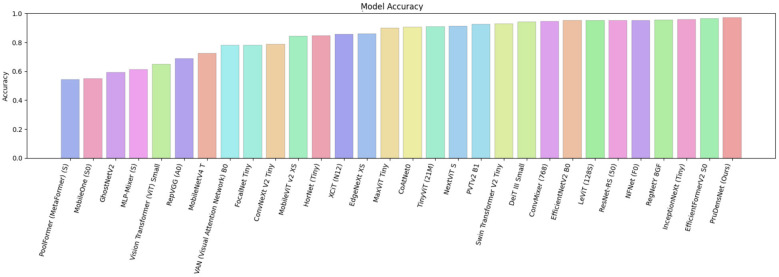
Model accuracy comparison, a bar chart showing the classification accuracy achieved by each evaluated architecture on the same test setting. Models are ordered from lowest to highest accuracy (left to right). PruDensNet (ours) attains the best accuracy among all compared methods.

**Figure 11:**
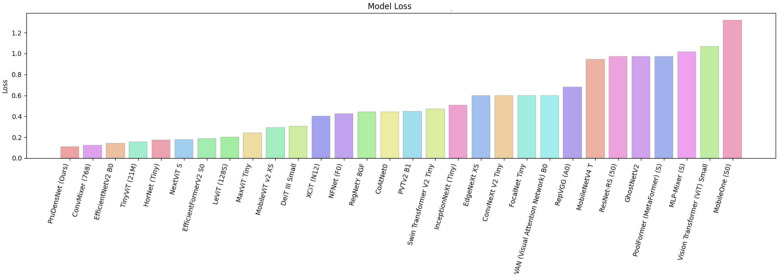
Model loss comparison, a bar chart showing the final loss value obtained by each evaluated architecture under the same experimental setup. Models are ordered from lowest to highest loss (left to right). PruDensNet (ours) achieves the lowest loss among all compared methods.

On the contrary, different baselines show significantly worse accuracy and increased loss, reflecting poorer generalization on the evaluated data. Figure 10 further compares model accuracies, ordering architectures from lowest to highest. Figure 11 displays the corresponding model loss values, where PruDensNet achieves the lowest loss. Overall, the joint trends across both figures show that PruDensNet (Ours) achieves the best trade-off among the methods compared with respect to existing CNN- and transformer-based approaches in terms of predicted correctness (accuracy) and optimization objective (loss).

**Figure 12:**
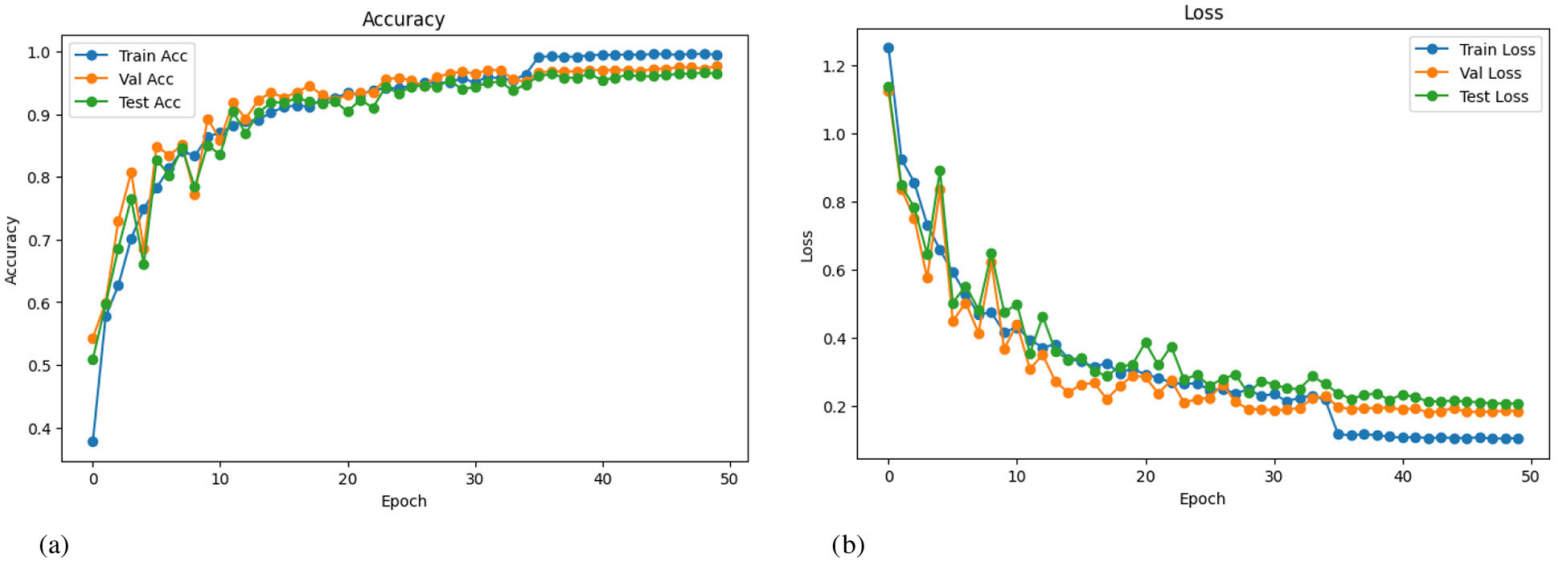
The graphs show training, validation, and testing performance over 50 epochs, which depict the model's learning behavior throughout training. The accuracy curve (top) shows both training and test accuracy improving steadily, indicating effective learning and minimal overfitting. The loss curve (bottom) demonstrates a consistent decline in both training and validation loss values, stabilizing near the end, which confirms convergence and strong generalization performance of the model. **(a)** The graph plots the accuracy of the model during training, validation, and testing across epochs. **(b)** The graph depicts the loss of values for training, validation, and testing across epochs.

In Figure 12, training, validation, and testing accuracy curves rise steadily over 50 epochs, while corresponding losses fall and stabilize. This pattern signals effective learning without notable overfitting. The late epoch indicates convergence under the chosen optimizer and schedule. Combined with the curriculum regularization, the curves suggest robust generalization. They also validate early stopping and checkpointing decisions described elsewhere. The figure visually closes the loop on the paper's training recipe and outcomes.

In Tables 5, 6, the per-class precision, recall, F1-score, and support are reported for the test and validation set. Glioma attains perfect precision and near-perfect recall, producing a very high F1. Meningioma and pituitary also show strong, balanced precision-recall, indicating consistent detection. The no-tumor class maintains high recall and F1, suggesting good specificity to healthy scans. These row-level results corroborate the aggregate accuracy in Table 2. The table thus demonstrates uniformly high class-wise performance.

**Table 5:** Validation-set classification report showing precision, recall, F1-score, and support for each class.

Class	Precision	Recall	F1-score	Support
glioma	0.9688	0.9841	0.9764	126
meningioma	0.9762	0.9462	0.9609	130
no_tumor	0.9881	0.9651	0.9765	86
pituitary	0.9640	0.9926	0.9781	135
Accuracy	–	–	0.9727	477
Macro avg	0.9743	0.9720	0.9730	477
Weighted avg	0.9729	0.9727	0.9727	477

**Table 6:** Test-set classification report showing precision, recall, F1-score, and support for each class.

Class	Precision	Recall	F1-score	Support
glioma	0.9844	0.9844	0.9844	128
meningioma	0.9375	0.9231	0.9302	130
no_tumor	0.9551	0.9770	0.9659	87
pituitary	0.9632	0.9632	0.9632	136
Accuracy	–	–	0.9605	481
Macro avg	0.9600	0.9619	0.9609	481
Weighted avg	0.9604	0.9605	0.9604	481

## 5 Discussion

PruDensNet demonstrates that a carefully engineered, depthwise-separable CNN augmented with lightweight channel-and-spatial attention can deliver state-of-the-art accuracy under tight parameter and memory budgets for four-class brain MRI classification. The observed test accuracy is 96.05% and validation accuracy 97.27% and uniformly high per-class precision, recall, F1 indicate that the combination of SE-CBAM-style gates, GELU activations, a compact GAP-Dense head, and a curriculum-regularized training recipe (annealed MixUp, CutMix, CLAHE, random erasing, dynamic label smoothing) yields tangible generalization benefits without incurring transformer-style complexity. Averaging predictions over five deterministic test-time transforms further stabilizes decisions. Importantly, the study's “parameter target padding” design enforces capacity-controlled comparisons (~1.46M trainables across models), strengthening the claim that gains arise from architectural choices rather than model size, while the leakage-aware pipeline (automatic class normalization, per-class near-duplicate filtering via aHash+SHA-1, and stratified file-level splits) mitigates common evaluation pitfalls in medical imaging benchmarks.

## 6 Limitations and future work

It is important to note the limitations within this study. One major limitation was the single dataset used for this analysis. This means that the results found in this study cannot be generalized to all scanners or facilities, nor can they be used to generalize about patients in different communities. Although the repeated-split evaluation demonstrated that the results were stable when evaluated, further validation on external datasets and/or multi-site will be required to establish that the method is indeed reliable and robust.

An additional limitation was that no patient identifiers were available in the dataset for analysis; therefore, we were unable to distinguish between patients on a patient-by-patient basis. Each patient had their own separate files; therefore, both file-level disjointness and duplicate patient removal were employed within the analysis of this study. Because of the possibility of some subtle data leakage in the dataset, there remains a possibility that some degree of overestimate could be made with regard to the actual performance of the method.

Future directions for this research include evaluation of the proposed method with a multi-institutional independent dataset and the acquisition of patient annotations that would allow for more rigorous evaluation of the method's performance. There will also be a need for more extensive investigation into how robust the model is with respect to distribution shifts, as well as its ability to be used in a “real-world” environment. All of these efforts will aid in furthering the overall potential of the method for use in clinical practice.

## 7 Conclusion

This study introduces PruDensNet, a parameter-efficient, attention-augmented depthwise-separable CNN with a reproducibility-focused training and evaluation pipeline that attains test accuracy 96.05%, Validation accuracy 97.27%, and outperforms representative CNN, transformer, and hybrid baselines under a matched ~1.4M-parameter constraint on a four-class brain MRI task. The contributions span architecture (SE+spatial attention with GELU and a compact head), methodology (capacity control via parameter padding; curriculum-style augmentation; mixed-precision optimization; five-view TTA), and data hygiene (automatic source discovery, class canonicalization, near-duplicate removal, stratified file-level splits). Together, these choices yield a deployable footprint (~5.6 MB) while preserving strong discriminative performance. Looking ahead, this study envisioned extending the framework to patient-wise and multi-center validations, incorporating explicit latency targets in model selection, and broadening evaluation to calibration metrics and clinically meaningful error analyses steps that would further strengthen readiness for translation to resource-constrained clinical environments.

## Data Availability

The original contributions presented in the study are included in the article/supplementary material, further inquiries can be directed to the corresponding author.
